# Isolated left ventricular non-compaction in Africa: elucidating myths

**Published:** 2013-06

**Authors:** Ferande Peters, Mohamed R Essop

## Dear Sir

We read with great interest the article by Falase *et al.* in the November 2012 issue of this journal.^1^ They stated that there are no documented cases of left ventricular non-compaction in Africa. Furthermore, they propose that this is due to a lack of awareness of this condition among African cardiologists. The purpose of this letter is to highlight that this statement is not entirely accurate.

Isolated left ventricular non-compaction has been the subject of several case reports^2^,^3^ occurring in individuals of sub-Saharan origin, as well as two large studies^4^,^6^ (Table 1). The first large study that documented this condition included 54 individuals, all of African origin with no evidence of any congenital or acquired heart conditions associated with the phenotype of left ventricular non-compaction.^4^

**Table 1.** Isolated Left Ventricular Non-Compaction In Individuals Of Sub-Saharan Origin

*Authors*	*Year of publication*	*Type of report*	*Sample size*	*Age (years)*	*Imaging technique*
Paule P, Braem L, Mioulet D, Jop B, Théron A, Gil JM, Héno P, Fourcade L^7^	2007	Case report	3	Range: 23–45	Echo and MRI
Massoure PL, Lamblin G, Bertani A, Eve O, Kaiser E^8^	2011	Case report	1	74	Echo
Peters F, dos Santos C, Essop R^2^	2011	Case report	1		Echo: Jenni criteria
Peters F, Khandheria BK, dos Santos C, Matioda H, Mogogane MT, Essop MR^3^	2012	Case report	2	35	Echo: Jenni criteria
Peters F, Khandheria BK, dos Santos C, Matioda H, Maharaj N, Libhaber E, Mamdoo F, Essop M^4^	2012	Large prospective series	54	Mean: 45.4 ± 13.1	Comprehensive criteria: combination of Jenni and Stolberger
Peters F, Khandheria BK, Libhaber E, Maharaj N, dos Santos C, Matioda H, Essop MR^6^	2013	Large prospective series	60	Mean: 47.01 ± 12.8	Comprehensive criteria: combination of Jenni and Stollberger

This study showed that using comprehensive echocardiographic criteria could distinguish between normal individuals and those with left ventricular non-compaction. Furthermore, the burden of right ventricular non-compaction and papillary muscle abnormality in this condition was highlighted for the first time, which corresponds to findings from the largest pathology study conducted thus far.^5^ We also recently described the myocardial mechanics of a cohort of subjects who fulfilled the echocardiographic criteria for isolated left ventricular non-compaction, with rigid body rotation occurring in 53% of subjects.^6^

It is important to note that currently, the only dedicated left ventricular non-compaction clinic in the southern hemisphere is based at the Chris Hani Baragwanath Hospital, where patients have now been followed up for up to 40 months. This centre has dealt with both national and international referrals, and offers echocardiographic techniques and CMR to aid diagnosis. In addition to standard medical therapy, patients with isolated left ventricular non-compaction have been treated with biventricular pacing and internal cardiac defibrillators in cases of refractory heart failure.

Isolated left ventricular myocardium is a condition encountered in clinical practice in Africa and needs to be identified and distinguished from acquired causes of left ventricular non-compaction. Long-term outcome and follow up have not been documented among patients of African origin and is the subject of future research initiatives.
